# Swimming Velocity Analysis Using Wearable Inertial Sensors and Speedometer: A Comparative Study

**DOI:** 10.3390/bioengineering11080757

**Published:** 2024-07-26

**Authors:** Leandro Vieira, Mário J. Costa, Catarina C. Santos, Francisco A. Ferreira, Ricardo J. Fernandes, Susana Soares, Márcio F. Goethel, João Paulo Vilas-Boas

**Affiliations:** 1 Centre of Research, Education, Innovation and Intervention in Sport (CIFI2D), Faculty of Sport and Porto Biomechanics Laboratory (LABIOMEP), University of Porto, 4200-450 Porto, Portugal; mjcosta@fade.up.pt (M.J.C.); cmsantos@fade.up.pt (C.C.S.); up202100039@up.pt (F.A.F.); ricfer@fade.up.pt (R.J.F.); susana@fade.up.pt (S.S.); gbiomech@fade.up.pt (M.F.G.); jpvb@fade.up.pt (J.P.V.-B.); 2 Department of Sport Sciences, Higher Institute of Educational Sciences of the Douro (ISCE-Douro), 4560-547 Penafiel, Portugal

**Keywords:** biomechanics, swimming, performance, breaststroke, speedometer, inertial sensors, intracycle velocity variation

## Abstract

The speedometer is widely used to evaluate swimming velocity but has some constraints. With the constant development of inertial units (IMUs), it is expected that they will become a good alternative to the speedometer. This study aimed to compare the data retrieved by an IMU and a speedometer when breaststroke is performed at maximum speed. Sixteen swimmers, nine males and seven females (20.3 ± 3.3 vs. 18.7 ± 1.1 years old, 65.8 ± 11.2 vs. 57.7 ± 9.1 kg of body mass and 1.75 ± 0.07 vs. 1.61 ± 0.10 m of height, respectively), performed 4 × 25 m of breaststroke sprint. They were equipped with an IMU fixed to the sacrum and with the line of an electromechanical speedometer (acquisition frequency of 50 Hz) fixed at the central point in the lumbar region. Statistical parametric mapping was used to compare the velocity curves, IBM SPSS was used for descriptive statistics and Bland–Altman plots were used for agreement of measurements. The results show that the IMU and speedometer do not show similar patterns, and the velocity values measured by the IMU are lower (*p* < 0.001). Bland–Altman plots presented a larger bias in terms of coefficient of variation and intracycle velocity variation. It can be concluded that IMUs and speedometers are not substitutes for each other as methods for evaluating intracycle velocity variations.

## 1. Introduction

Swimming is characterized by the intermittent application of propulsive force to overcome hydrodynamic drag, which cyclically changes its intensity [1]. Consequently, the movement of the swimmer`s body in the water is not uniform and results in an intracycle velocity variation (IVV) [1,2]. So, the evaluation of the IVV can be used as a tool for evaluating the swimmer’s technique, allowing the determination of other kinematic measures such as accelerations (resulting from the prevalence of propulsive actions and decelerations), the shape and the actions of the swimmer’s body and jerkiness in the acceleration profile over time, as well as body position changes and displacement.

Kinematical evaluation in sports can be achieved using different methods: (i) video cameras, using two- (2D) and three-dimensional (3D) procedures, with and without body markers [2,3], (ii) speedometer [4], (iii) GPS [5], (iv) radar [6], (v) infrared marker tracking [7], and (vi) accelerometers or inertial measurement units (IMUs) [8,9]. There is general consensus on the advantages and disadvantages of each one of these methods, mostly related to the cost of equipment, complexity of the set-up and/or time required for processing the information. Video analyses require a computational (off-line) effort, favoring a delay in providing quantitative information [10] that is not in line with coaches’ expectations. The speedometer is a widely used device [4,11] and has its own constraints, mostly related to the cable connection (not allowing performing turns) and only allowing the measuring of one swimmer at a time. Therefore, the use of IMUs has become a relevant solution for characterizing quantitative human movement and analyzing swimming performance [10].

IMUs are devices that incorporate accelerometers to measure 3D accelerations, gyroscopes to evaluate 3D angular velocity and magnetometers to assess the magnetic field or magnetic dipole moment. IMUs have already been used in swimming and seem to provide a reliable solution for extracting kinetic- and kinematic-related variables [12]. Recent developments concerning IMU dimensions, reliability and price have made this piece of equipment a promising option for swimmer evaluation, with the potential to provide fast and easy-to-use information on detailed performance-related metrics [13]. It is possible to provide, in real-time, the cycle rate and lap times [14]. Although some questions persist regarding the orientation of the IMU sensors [15,16], and most existing studies have been carried out on the front crawl, IMUs are welcome devices because they allow kinematic variables to be extracted, making them a powerful tool for swimming analysis [17,18]. However, swimming speed can be a conditioning factor in the accuracy of the data collected [19].

The aim of the current study was to compare the data extracted from an IMU and a speedometer during breaststroke performed at maximal intensity; it being hypothesized that there will be a high level of agreement between devices. For that purpose, breaststroke intracycle velocity variation was evaluated using both devices and compared to the values obtained using Statistical Parametric Mapping (based on an independent parametric *t*-test and the Bland–Altman plots). We expected that the IMU might replace the speedometer in assessing swimming velocity without the limitations imposed by the cable connection and allow the collection of data from several swimmers simultaneously. If speedometers could be replaced by IMUs when assessing swimmers’ speed, this would permit an increase in swimmer monitoring frequency. This would impact positively swimmers’ technique and, consequently, raise their efficiency, concurrently with allowing the identification of technical errors that might also help prevent injuries with evident benefits to practitioners’ health.

## 2. Materials and Methods

### 2.1. Sample

Sixteen swimmers, nine males and seven females (20.3 ± 3.3 vs. 18.7 ± 1.1 years old, 65.8 ± 11.2 vs. 57.7 ± 9.1 kg of body mass and 1.75 ± 0.07 vs. 1.61 ± 0.10 m of height, respectively), participated in this study. The inclusion criteria were to have a minimum competitive experience of three years, a minimum classification of level two [20] and a specialization in breaststroke, as well as to be absent of injury in the six months prior to the data collection. Swimmers participated in 6.4 ± 2.6 training sessions per week (with a volume of 4100 ± 1300 m per session) and attained a performance level of 386 ± 86 points in the 100 m breaststroke event according to the World Aquatics Point Scoring. All swimmers (or legal guardians) were informed of the experiment’s benefits and risks before giving written informed consent for participation. The study was conducted according to the Helsinki Declaration.

### 2.2. Study Design

The experimental setup was set on a short-course indoor swimming pool with a water temperature of 25 °C, an air temperature of 23 °C and 60% humidity. The participants were initially tested for anthropometric measures using only their textile swimming suits and caps. Body height and mass were measured to the nearest 0.1 cm and 0.1 kg, respectively, using a portable stadiometer (SECA, 242, Hamburg, Germany) and a portable scale (TANITA, BC-730, Amsterdam, The Netherlands). Then, the participants were asked to perform a standard warm-up composed of 100 m of front crawl and 100 m of breaststroke at low intensity, plus 4 × 50 m of breaststroke at an increasing pace. For the in-water testing, swimmers were randomly assigned to perform 4 × 25 m of breaststroke at maximal intensity, starting with a wall push-off after an auditory signal, with 2 min of rest interval between trials.

Swimmers were instrumented with one IMU (GT9XActiGraph Link, Pensacola, FL, USA) composed of 3D accelerometer, gyroscope and magnetometer. The accelerometer and gyroscope data were sampled at 100 Hz frequency using a full-scale set at ±8 g and ±2000 deg·s^−1^ (respectively). The IMU was attached to the swimmer’s sacrum, waterproofed (inserted and sealed in a condom) and carefully positioned to allow the best coincidence to the global reference coordinate system (X, Y and Z axes: horizontal, lateromedial and vertical). A validated speedometer (with a 50 Hz acquisition frequency [21]) was fixed to a starting block and connected to the swimmer with a strap at the waist (as a satisfactory representation of the center of mass position [22,23,24]). The IMU device was calibrated by attaching it to the speedometer cable and performing a series of displacements. The relation between the velocity measured by the speedometer and the IMU data was used for the calibration mentioned above.

The linear acceleration and angular velocity data were initially filtered with a fourth-order Butterworth high-pass filter with a 1 Hz cut-off frequency to remove high-frequency noise. Then, the angular displacement data around the X, Y and Z axes were obtained through cumulative trapezoidal numerical integration of the angular velocity curves around these axes [25]. Afterward, the linear acceleration data were rotated [26] to precisely assume the global coordinate system orientation, establishing anteroposterior, mediolateral and vertical axes in relation to the swimming pool. The linear velocity curve in the anteroposterior axis was obtained through cumulative trapezoidal numerical integration of the linear acceleration curve along the anteroposterior axis. The analysis of the velocity variations during breaststroke was made using the coefficient of variation (CV = SD/mean) and IVV using the equation [2,27]:

$$IVV=\frac{\left(vmax\_LL-vmin\_LL+Vmax\_UL-vmin\_T\right)}{v}$$

where the vmax_LL is the maximum velocity achieved during the lower limbs’ propulsion, vmin_LL is the first minimum velocity following upper and lower limbs recovery (the beginning of lower limbs propulsion), vmax_UL is the maximum velocity registered during the upper limbs’ propulsion, vmin_T is the minimum velocity observed during the transition between lower and upper limbs propulsion and v is the mean swimming velocity during the cycle.

### 2.3. Statistical Analysis

The Statistical Parametric Mapping, based on an independent *t*-test, was computed using the SPM1D package (version 0.4.3, https://spm1d.org/, accessed on 1 June 2023) on MATLAB R2022a with α = 0.05 to compare the velocity curves profiles (normalized to 101 data points) of both devices. Using IBM SPSS (version 28.0), we checked the normality of the distribution with the Kolmogorov–Smirnov test and calculated mean, standard deviation and P for parametric data treatment and median, interquartile range and P for non-parametric data. The Bland–Altman plot [28] was applied using BA Plotter [29] according to the guidelines [30] to quantify the agreement between two quantitative measurements by determining the bias (or mean difference) and limits of agreement as a measure of accuracy and precision (respectively). The mean of the two measurements was plotted against their difference, with 95% of the differences expected to lie within the limits of agreement (mean [1.96 SD]) and respective 95% confidence interval (CI). The CI of the bias and the limits of agreements illustrates the magnitude of the systematic error and an estimation of the extent of the possible sampling error (respectively) [28,30]. Prisma GraphPad Prism 10 (Dotmatics, Bishops Stortford, UK) was also used for analyzing the slope of the regression line between the two devices to check for proportional error.

## 3. Results

**Figure 1:**
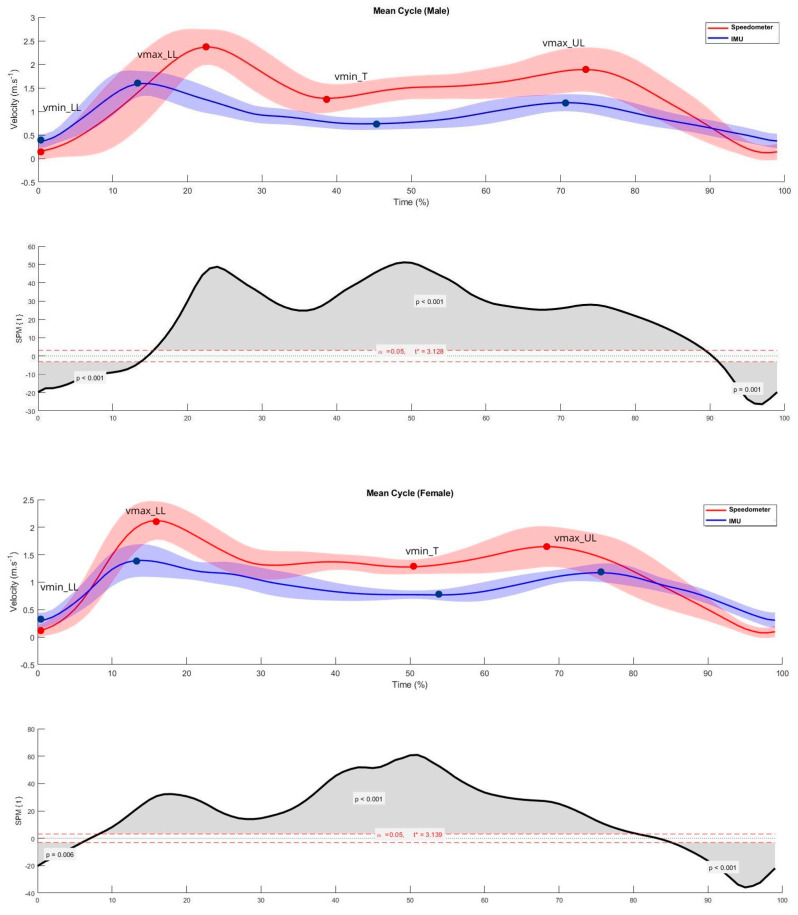
Average male and female breaststroke cycle and respective Statistical Parametric Mapping representation (upper and lower panels, respectively). In red and marked with an asterisk is the value of the t statistic for the upper and lower significance thresholds.

**Figure 2:**
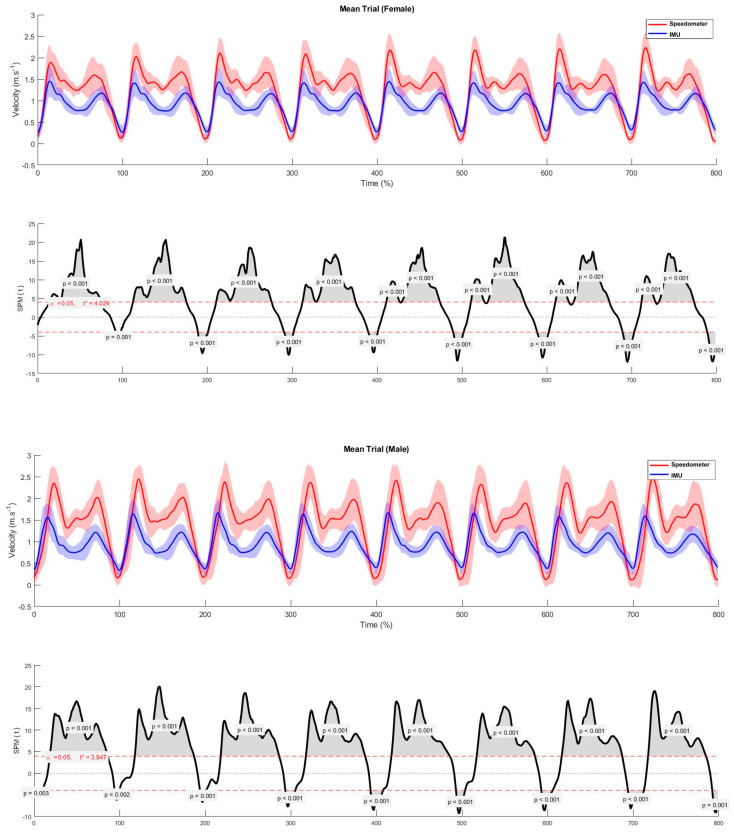
Set of eight average breaststroke swimming cycles (male and female) and respective Statistical Parametric Mapping to compare measurements (upper and lower panels, respectively). In red and marked with an asterisk is the value of the t statistic for the upper and lower significance thresholds. The time elapsed in the different cycles is standardized, expressing a temporal uniformity in percentage (Time%). Eight mean cycles, being 100% to each, correspond to 800% total time.

Figure 1 represents one male and one female average breaststroke cycles from the 512 cycles analyzed and Figure 2 represents the average of each one of the first eight breaststroke cycles after the swimmer’s head breaks the water surface, for males and females. The beginning of each cycle was considered at the lowest point of the velocity time series, i.e., immediately before the propulsive action of the lower limbs.

From a qualitative curve-profile interpretation, there is a “macro” trend to the two main maximums, which are out of phase in time and also do not agree in absolute velocity values. There were notable differences regarding the velocity values measured by the two devices, with the speedometer showing higher values than the IMU during most of the cycle. However, there seems to be a tendency for IMU and speedometer values to provide similar data at lower speeds, such as at the beginning of the lower limbs’ propulsive action and the end of the upper and lower limbs’ recovery. Specifically, in the average male and female curves, there are differences between measuring systems in the following ranges (0–13.7%; 15.5–89.3% and 91.1–99.0% vs. 0–5.9%; 8.2–80.5% and 5.1–99.0%). The same trend (similar values at lower speeds) is observed in the average curve of the first eight cycles of each swimmer after the head breaks the surface of the water.

**Figure 3:**
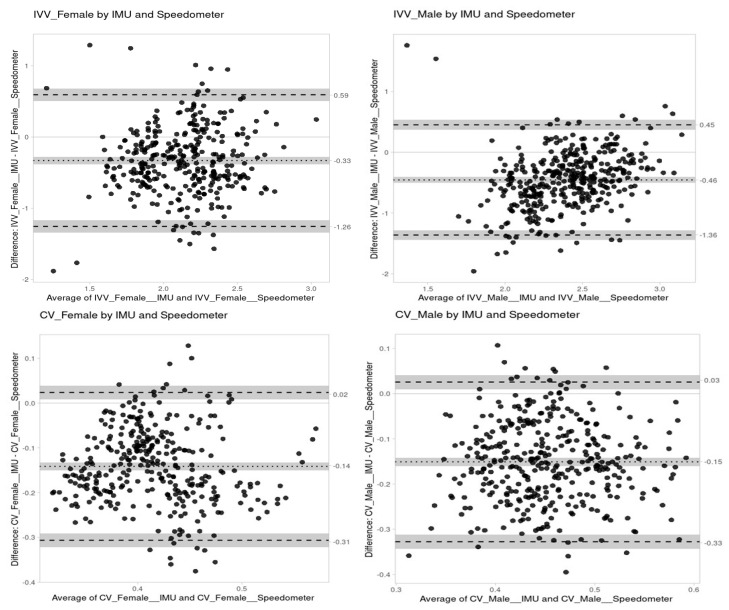
Agreement between IMU and speedometer data for IVV female, IVV male and CV female, CV male (upper and lower panels, respectively).

Table 1 presents a comparison of the IVV and CV obtained by the speedometer and the IMU in males and females. The data distributions were found to be normal for the IVV obtained both from the speedometer and IMU and for the CV of data obtained from the IMU. Figure 3 presents Bland–Altman Plots dissecting the agreement between IVV and CV using the velocity values collected by the IMU and speedometer. In the BA plots, the mean of the differences is represented by the dotted line, the limits of agreement by the dashed lines and the confidence intervals by the shading. Female IVV showed a bias of -0.33 and, despite some outliers and some data points out of the limits of agreement, a consistent distribution between the limits of agreement (−1.26 to 0.59) and respective 95% CI (96.24% of all data points laid inside the confidence bounds) was observed. No proportional error (*p* > 0.05) was noted.

Female CV bias was −0.14 and, likewise, female IVV also observed a consistent distribution between the limits of agreement (−0.31 to 0.02) and respective 95% CI (96.42% of all data points laid inside the confidence bounds). Male CV showed −0.15 of bias and, despite some outliers, a consistent distribution was observed between the limits of agreement (−0.33 to 0.03) and respective 95% CI (97.33% of all data points lay inside the confidence bounds). Comparing the male IVV obtained by the two methods, we found a bias of −0.46 and, despite some outliers, a consistent distribution was observed between the limits of agreement (−1.36 to 0.45) and respective 95% CI. A slight trend in the distribution was observed, although very slight, for a lower bias as IVV increases.

## 4. Discussion

Data differences in the magnitude of velocity values obtained by the IMU and speedometer were observed, despite them both pointing to patterns characterized by two main velocity peaks, as described before [8,31,32]. However, the time stamp of these peaks, and corresponding intermediate values, did not match, not allowing similar interpretations regarding the time partition of the breaststroke technique in different phases. This seems to conflict with the findings of a previous study, where the 3D wrist trajectory obtained by an inertial system was considered to allow accurate and complete identification of front crawl phases [33]. Furthermore, if the intracycle velocity variation is considered, the curves produced by both systems are different for almost the entire cycle; it being possible to observe that there is a tendency for the measured values to be more similar at the beginning and end of the cycle considered. In fact, the start of the lower limb action and the end of the recovery is a phase where the magnitude of the velocity is lower. At the phases of greatest velocity (lower and upper limb propulsive actions), the measurements obtained by the two methods were significantly and expressively different.

Next, we tried to verify if the observed differences between the IMU and speedometer also extend to both IVV and CV, as metrics commonly associated with the overall quality of the swimming action. Most of the Bland–Altman plot data points are below zero and all biases are negative, meaning that there is a tendency for the IVV and CV values obtained through the velocity measures accessing from the IMU to be lower than the values of speed variation obtained from the speedometer records, as verified through the analysis of the Statistical Parametric Mapping instantaneous values of velocity. It was found that, although the percentage of values outside the confidence intervals was not high, the bias for CV is very large (14 and 15% for the CV of women and men, respectively, while the IVV showed a bias of 0.33 and 0.46 for women and men, respectively). In this sense, the use of an IMU to estimate CV or IVV is not an alternative to the data presented by the speedometer.

According to the literature, some authors consider that one single IMU placed on the sacrum can be used to measure the velocity [34], while others consider that it is not sufficient to determine or infer the cycle phases with accuracy [35]. It is also emphasized in some studies that wireless data transfer is a necessity, but signal loss needs to be minimized [36]. The need for a compromise between the potential of technology and practicality in the field is also suggested [37] regarding the necessity to solve the issues related to the lack of adequate standardization of data acquisition and tools for subsequent analysis, so that their use by coaches may be increased. In recent years, we have seen an increase in the number of studies validating inertial sensors [15,38,39], and with many uses of IMUs, like to identify cycle phases [40], swimming techniques [41,42], swim turns, underwater gliding and clean swimming [43], even the quantification of energy expenditure [44]. Accordingly, we recognize that IMU technology has developed and the devices are increasingly smaller in size. Depending on the number in simultaneous use and the position in which they are placed, they can be used without causing embarrassment to the swimmer and allow a lot of information to be collected at the same time. They also make it possible to assess a group of swimmers. However, they are not yet an alternative to the speedometer when it comes to assessing intracycle velocity variation. So, further studies should be carried out in this area, which will enable the development of algorithms and other procedures leading to the collection of increasingly accurate and easy-to-use information. This aspect is of greater importance when the velocity values referenced to the IMU are not obtained directly but calculated. In addition, it will be necessary to work on optimizing this process to avoid bias.

## 5. Conclusions

It can be concluded that in the velocity curves acquired using an IMU and a speedometer during breaststroke swimming, there is an underestimation of velocity values by the IMU compared to the values of the speedometer, which can be seen mostly at higher swimming velocities. IMUs and speedometers do not show similar patterns and show significant differences. They are only similar in showing a “macro” trend to two main maximums, but the respective maximums are out of phase in time and absolute velocity values. So, IMUs and speedometers are not substitutes for each other for velocity measurement.

## Figures and Tables

**Table 1:** Comparison of IVV and CV obtained by different devices: values are mean and standard deviation and P for parametric data treatment and median, interquartile range and P for non-parametric data.

	Females			Males		
	Speedometer	IMU	*p*	Speedometer	IMU	*p*
**IVV**	2.26 (0.51)	1.96 (0.55)	<0.01	2.60 ± 0.28	2.12 ± 0.39	<0.01
**CV**	0.47 (0.10)	0.35 (0.08)	<0.01	0.52 (0.10)	0.37 (0.10)	<0.01

## Data Availability

Data are contained within the article.
